# Preventive health care among HIV positive women in a Utah HIV/AIDS clinic: a retrospective cohort study

**DOI:** 10.1186/1472-6874-14-37

**Published:** 2014-03-04

**Authors:** Sara E Simonsen, Deanna Kepka, Joan Thompson, Echo L Warner, Maggie Snyder, Kristen M Ries

**Affiliations:** 1 Department of Family and Preventive Medicine, Division of Public Health, University of Utah, 375 Chipeta Way, Suite A, Salt Lake City, UT 84108, USA; 2 College of Nursing, University of Utah, Huntsman Cancer Institute; 2000 Circle of Hope, Salt Lake City, UT 84112, USA; 3 Department of Internal Medicine, Division of Infectious Diseases, University of Utah, 30 North 1900 East, Room 4B319, Salt Lake City, UT 84112, USA; 4 Cancer Control and Population Sciences, Huntsman Cancer Institute; 2000 Circle of Hope, Salt Lake City, UT 84112, USA

**Keywords:** HIV/AIDS, Women, Preventive health services, Cancer screening, Safe-sex counseling, Sexually transmitted infection testing

## Abstract

**Background:**

Despite evidence that HIV positive women may suffer higher rates of heart disease, diabetes, human papillomavirus infection, and some types of cancer, the provision of preventive health services to HIV positive women is unknown. Preventive health services recommended for such women include breast, colorectal and cervical cancer screening, sexually transmitted infection (STI) testing, vaccinations, and patient counseling on a number of issues including sexual behaviors.

**Methods:**

This retrospective cohort study utilized medical record reviews of 192 HIV positive women who were patients at the University of Utah Infectious Diseases Clinic in 2009. Medical records were reviewed for all encounters during 2009 using a standardized data collection form; data were collected on patient demographics and a variety of preventive health services. Chi squared tests were used to assess receipt of preventive health services by demographic factors, and multivariable logistic regression was used to determine predictors of receiving select services.

**Results:**

The most commonly recorded preventive services included blood pressure screening, screening for Hepatitis A and B, Tetanus-Diphtheria-Pertussis vaccination, Pneumococcal pneumonia vaccination, substance abuse screening, and mental health screening. STI testing and safe sex counseling were documented in the medical records of only 37% and 33.9% of women, respectively. Documentation of cancer screening was also low, with cervical cancer screening documented for 56.8% of women, mammography for 65% (N = 26/40) of women, and colorectal cancer screening for 10% (N = 4/40) of women, where indicated. In multivariable models, women with private health insurance were less likely to have documented STI testing (OR 0.20; 95% CI 0.08 - 0.52), and, Hispanic women were less likely to have documented safe-sex counseling (OR 0.26; 95% CI 0.07 - 0.94).

**Conclusions:**

HIV/AIDS providers should focus on the needs of all women for preventive care services, including those with fewer socio-demographic risk factors (i.e., insured, stable housing etc.). In addition, failure to provide STI testing, cancer screening, or safe sex counseling to all patients represents a missed opportunity for provision of services that are important from both a clinical and public health perspective.

## Background

Since the advent of highly active anti-retroviral therapy (HAART), the average life expectancy for HIV positive individuals has increased, and patients with HIV are experiencing lower rates of AIDS and AIDS-defining illnesses. However, as the mortality directly associated with HIV/AIDS has decreased, mortality rates from non-HIV-related conditions such as cardiac disease and non-AIDS-related cancers have increased [1]. While rates of breast, colon, and prostate cancers are similar in HIV positive and HIV negative individuals, rates of dyslipidemia and diabetes are higher among HIV positive patients [1]. Additionally, many HAART drugs such as protease inhibitors, are associated with increased risk for certain comorbid conditions such as cardiovascular disease and diabetes [2,3]. As the HIV positive population ages, it is important that clinicians provide routine, preventive health care and screening for conditions such as colon cancer, hypertension, and dyslipidemia to these patients [1,4].

In 2009, the HIV Medicine Association (HIVMA) of the Infectious Diseases Society of America (IDSA) announced new guidelines for providing preventive healthcare for HIV positive individuals [5]. In accordance with the United States Preventive Services Task Force (USPSTF), the HIVMA recommends that all HIV positive individuals age 50 and older have routine colorectal and breast cancer screening [6,7]. In addition, STI testing is recommended annually for syphilis, gonorrhea and chlamydia [6]. Regular patient counseling is recommended regarding sexual behavior and drug use as well as education about healthy diet, weight reduction, smoking cessation, and seat belt use. Lastly, many vaccinations are recommended for HIV positive individuals, including inactivated annual influenza vaccine [6].

HIV positive women have unique preventive health care needs, including breast, colorectal, and cervical cancer screening, counseling about family planning and contraceptive use, and pregnancy care, as applicable [4]. USPSTF and HIVMA guidelines include annual breast and colorectal cancer screeing for women age 50 and older and cervical cancer screening starting at age 21, conducted annually if two normal Pap tests are documented during the first year after HIV diagnosis [6] and the CD4 count is >200 cells/μL and increasing to every 6 months if the CD4 count is <200 cells/μL [8]. Attention to preventive health care in this population is critical, as evidence suggests that they may be less likely to receive breast cancer screening then their HIV negative peers, [9] are at greater risk for sexually transmitted infections (STI), [10] and are more likely to be infected with human papillomavirus (HPV) and to develop persistent HPV infections [11]. It has been estimated that as many as 60% of HIV positive women experience co-infection with HPV [12]. HIV positive women are at increased risk for cervical dysplasia, [13] and more severe cervical cancer when compared to the general population, with a lower average age at infection and a considerably shorter developmental period [14]. HIV positive women are also at higher risk for other comorbidities such as coronary heart disease and metabolic syndrome, [15] diabetes and insulin resistance, [16] and other cancers [17]. The high burden of comorbidities among HIV positive women requires appropriate and timely preventive health care.

Unfortunately, many factors serve as barriers to care for HIV positive women, including socioeconomic and structural barriers, poverty, cultural inequities, sexual violence, physiologic differences, lack of awareness of HIV status, and the ongoing stigma of being HIV positive [18]. HIV positive women are at increased risk for social/emotional issues, [19] sexual violence, [20] trauma, substance abuse and addiction, [19] homelessness, and abuse [21]. Over half of HIV positive women report experiencing some form of intimate partner violence in adulthood [20]. In addition, HIV positive women report poorer mental health than HIV negative women, [22] and HIV positive women with depressive symptoms are more likely to report frequent and severe abuse [23]. HIV positive women who are racial/ethnic minorities are at especially high risk for disparities in health care access and quality, exacerbated by language and cultural barriers [18,24,25]. Non-English speaking women are less likely to receive preventive services, [26] and although professional interpreters improve the quality of care for such patients, the use of interpreters in clinical settings is vastly underutilized [27-29]. Effective patient-provider communication is key to improving quality of care, [30] including provision of recommended preventive services, but can be a challenge. Barriers to effective patient-provider communication among women with HIV, particularly racial/ethnic minorities, may impact the quality of the preventive healthcare services they receive [31,32].

Despite improved accessibility to antiretroviral treatments, and improved life expectancy among HIV positive women in care, little research has been done to document the extent to which such women are receiving appropriate preventive health care in accordance with recommended guidelines. In this study, we conducted a medical records review of all HIV positive women seen in a Utah HIV/AIDS specialty clinic to describe the demographic characteristics of these women, identify gaps in the services they received, and evaluate predictors of receipt of preventive health services. This review was done in 2009 to correspond with the 2009 HIVMA/IDSA guidelines for preventive healthcare for individuals with HIV/AIDS.

## Methods

### Data collection & participants

This retrospective cohort study utilized medical record reviews of HIV positive women who were patients at the University of Utah Infectious Diseases Clinic (Clinic 1A) in 2009. It is estimated that Clinic 1A serves an estimated 70-80% of all HIV/AIDS patients in care in Utah. Female patients comprised 16% of the clinic population in 2009 and 13% of individuals with HIV/AIDS in Utah [33]. Women seen in this clinic were similar demographically to all HIV/AIDS patients in Utah. All HIV positive women seen at least once at Clinic 1A between January 1, 2009 and December 31, 2009 (n = 192) were included in the study. Data abstractors used a standardized data collection form and entered data using protocols developed through a consensus process. Both electronic and paper medical records were reviewed for specific information about patient demographics and health screenings (blood pressure, lipid panel, cervical cancer screening, breast cancer screening, colorectal cancer screening, mental health screening, and substance abuse screening). Mammography and colorectal cancer screening were indicated for women ages 50–75 (N = 40) in accordance with USPSTF guidelines. Medical records were also reviewed for documentation of safe sex counseling, vaccinations, sexually transmitted infection (STI) testing, and tuberculosis (TB) testing. Complete STI testing was defined as receipt of syphilis, gonorrhea, and chlamydia screening. Housing status, obtained from the medical record, was defined as stable, homeless, transient or subsidized. Lastly, the records were reviewed for comorbidities listed in the patient’s “problem list”, medications used, adherence (missed visits, missed doses of HAART), and referrals for eye exams, dental exams, and case management. The study was approved by the University of Utah Institutional Review Board.

### Statistical analysis

Data were transferred into Stata 12.1 (College Station, Texas) for data analysis. Descriptive statistics were calculated to describe the demographic characteristics of the women (Table 1) and the preventive health services they received, with a focus on breast, cervical and colorectal cancer screening; mental health and substance abuse (including alcohol and drugs) screening; eye and dental exam referral; STI testing including syphilis, gonorrhea, and chlamydia; safe sex counseling; annual influenza vaccination; and tuberculosis (TB) testing. For bivariate analyses and multivariate regressions, we created dichotomous variables for each of the preventive health services in Table 2 (services not received/not recorded vs. service received). Because we were rarely able to determine from the medical record when a service was offered and declined (or not indicated) vs. not offered, we combined these responses with the exception of screenings indicated by age. For screenings indicated by age, we limited analyses to women in the appropriate age ranges. We used bivariate analyses to identify differences in the receipt of preventive health services, including: cervical cancer screening, mammography, STI testing, safe sex counseling, influenza vaccination, TB testing, mental health screening, and substance abuse screening. Each preventive health service was evaluated by: race, Hispanic ethnicity, U.S. citizenship, housing status, insurance status, and language. Chi-squared statistics were used to evaluate the differences in screening behaviors by select demographic characteristics including race (White vs. Non-White), ethnicity (Hispanic vs. Non-Hispanic), citizenship (U.S. citizen vs. other), housing status (stable vs. other), insurance status (private vs. other), and language (English vs. other). Finally, multivariable-adjusted odds ratios were calculated for preventive health service variables with significant between-group differences in more than one chi-squared analysis. These included STI testing and safe sex counseling. Covariates of interest in these models included race, ethnicity, immigration status, housing status, insurance, and language. All statistical tests were two sided, and an alpha level of 0.05 was used to determine statistical significance.

**Table 1:** Demographics of HIV positive women seen at Clinic 1A in 2009

**N = 192**	**N**	**%**
**Current age**^**1**^		
18-29	25	13.0
30-39	72	37.5
40-49	52	27.1
50-59	32	16.7
≥60	9	4.7
**Race**^**2**^		
Caucasian	131	68.2
African	46	24.0
Other	11	5.7
**Ethnicity**^**3**^		
Hispanic	41	21.3
Non-Hispanic	145	75.5
**Insurance status**^**4**^		
Private insurance	61	31.8
Medicaid/Medicare	59	30.7
Primary Care Alliance (Ryan White Part C Program)	61	31.8
Other	10	5.2
**Immigrant status**^**5**^		
US citizen	118	61.5
Other	47	24.5
**Primary language**^**6**^		
English	121	63.0
Spanish	24	12.5
Other	42	21.9
**Housing status**^**7**^		
Stable	137	71.3
Homeless/transient/subsidized	38	19.8
**HIV/AIDS status**^**8**^		
HIV, not AIDS	100	52.1
AIDS	88	45.8

^1^Current age not recorded/missing for N = 2.

^2^Race not recorded/missing for N = 4.

^3^Ethnicity not recorded/missing for N = 6.

^4^Insurance status not recorded/missing for N = 1.

^5^Immigrant status not recorded/missing for N = 27.

^6^Primary language not recorded/missing for N = 5.

^7^Housing status not recorded/missing for N = 17.

^8^HIV/AIDS Status not recorded/missing for N = 4.

**Table 2:** **Receipt of preventive services among HIV positive women seen at Clinic 1A in 2009**^**1**^

**N = 192 unless otherwise indicated**	**N**	**%**
**Cervical cancer screening**^**2**^		
Yes	109	56.8
No	18	9.4
Not indicated^4^	5	2.6
Not recorded/missing	60	31.2
**Mammogram among women age 50–75 (N = 40)**^**3**^		
Yes	26	65.0
No	2	5.0
Not indicated^4^	0	0
Not recorded/missing	12	30.0
**Colorectal cancer screening among women age 50–75 (N = 40)**^**3**^		
Yes	4	10.0
No	3	7.5
Not indicated^4^	1	2.5
Not recorded/missing	32	80.0
**Mental health screening**		
Yes	159	82.8
No	0	0
Not indicated^4^	0	0
Not recorded/missing	33	17.2
**Substance abuse screening**		
Yes	176	91.7
No	0	0
Not indicated^4^	0	0
Not recorded/missing	16	8.3
**Eye exam referral**		
Yes	49	25.5
No	0	0
Not indicated^4^	0	0
Not recorded/missing	143	74.5
**Dental exam referral**		
Yes	62	32.3
No	0	0
Not indicated^4^	0	0
Not recorded/missing	130	67.7
**Syphilis testing**		
Yes	133	69.3
No	0	0
Not indicated^4^	0	0
Not recorded/missing	59	30.7
**Gonorrhea testing**		
Yes	86	44.8
No	0	0
Not indicated^4^	0	0
Not recorded/missing	106	55.2
**Chlamydia testing**		
Yes	89	46.3
No	0	0
Not indicated^4^	0	0
Not recorded/missing	103	53.7
**Tested for syphilis, gonorrhea, & chlamydia**		
Yes	71	37.0
No	121	63.0
**Safe sex counseling**		
Yes	65	33.9
No	0	0
Not indicated^4^	3	1.6
Not recorded/missing	124	64.6
**Annual influenza vaccine**		
Yes	138	71.9
No	8	4.1
Not indicated^4^	0	0
Not recorded/missing	46	24.0
**TB testing**		
Yes	121	63.0
No	4	2.1
Not indicated^4^	26	13.5
Not recorded/missing	41	21.3

^1^For bivariate analyses and multivariate regressions we created dichotomous variables for each of the preventive health services in Table 2 (services not received/not recorded/missing vs. service received).

^2^No age cut off for women who are immunocompromised (including HIV positive) according to USPSTF recommendations.

^3^Excludes N = 152 participants who were outside of the USPSTF recommended age range for mammogram and colorectal cancer screening (<50 years or >75 years).

^4^Not indicated means the medical record has a note about a procedure not being done and why it was not done.

## Results

### Demographics

Table 1 displays select demographic characteristics of the participants. There were 192 HIV positive women seen in Clinic 1A in 2009. Of these, 52.1% had HIV and 45.8% had AIDS. Similar to the demographics of all HIV/AIDS patients in Utah, the majority of women in the study were Caucasian (68.2%) or of African descent (24%). Over three quarters of women (75.5%) were non-Hispanic and 61.5% were U.S. citizens. Countries of origin included: Angola, Burma, Burundi, Congo, Ethiopia, Ghana, Liberia, Rwanda, Sudan, South Africa, Tanzania, Togo, Mexico, Argentina, El Salvador, Haiti, Honduras, Venezuela Thailand, and Russia. Sixty-six (34.4%) women reported a primary language other than English. Languages included: Spanish, Bassa, Amharic, Arabic, Gromo, Burmese, Ewe, French, Hatian, Italian, Kingarwanda, Swahili, Linggla, Kirundi, Mali, Mende, Vai, Pakistani, Portugese, Russian, Thai, Vietnamese, and Zulu. The women had a variety of contextual and behavioral risk factors, with only 31.8% reporting private health insurance, 19.8% reporting being homeless or living in transient or subsidized housing, 38.5% having a mental health issue and 21% reporting substance abuse including abuse of alcohol or drugs.

### Receipt of preventive health services

The most commonly recorded preventive health services received by HIV positive women included blood pressure screening, screening for Hepatitis A and B, Tetanus-Diphtheria-Pertussis vaccination, Pneumococcal pneumonia vaccination, substance abuse screening, and mental health screening. All of these preventive health services were received by at least 75% of women (data not shown). However, only 37% of women received complete STI testing including syphilis, gonorrhea, and chlamydia testing, and 33.9% had safe sex counseling documented in their medical record during the last year. Additionally, 56.8% of women received cervical cancer screening, 65% (N=26/40) of women age 50 or older received a mammogram and 10% (N=4/40) of women age 50 or older received colorectal cancer screening. Less than one-third of women received a referral for a dental or eye exam. Data on select preventive health services are shown in Table 2.

### Factors related to receipt of preventive health services

Non-white women were significantly *more* likely to have STI testing (p = 0.008) and safe sex counseling (p=0.001) documented in their medical records, while Hispanic women, English speaking women, and those with U.S. citizenship were *less* likely to have documented safe sex counseling (p = 0.002, 0.017, and 0.004, respectively). Women with private insurance were also *less* likely to have documented STI testing (p < 0.001) or cervical cancer screening (p = 0.025). However, women in stable housing situations were *more* likely to have mental health screening (p = 0.020), and substance abuse screening (p = 0.026) documented in their medical records (Table 3). No significant differences in services were observed for women with substance abuse issues, comorbidities, or mental health issues (data not shown).

**Table 3:** **Receipt of select preventive health care services, stratified by demographic characteristics**^**1**^

	**Pap testing**		**Mammogram**		**STI testing**^**2**^		**Safe sex counseling**		**Influenza vaccine**		**TB testing**		**Mental health screening**		**Substance abuse**	
	**p =**		**p =**		**p =**		**p =**		**p =**		**p =**		**p =**		**p =**	
	**N**	**%**	**N**	**%**	**N**	**%**	**N**	**%**	**N**	**%**	**N**	**%**	**N**	**%**	**N**	**%**
**Race**	*0.682*		*0.979*		***0.008***		***0.001***		*0.225*		*0.182*		*0.472*		*0.151*	
White	73	57.5	17	42.5	40	30.5	33	25.6	95	96.0	82	70.7	107	81.7	123	93.9
Non-white	34	60.7	9	42.9	29	50.9	31	55.4	40	90.9	38	80.9	49	86.0	50	87.7
**Ethnicity**	*0.358*		*0.241*		*0.772*		***0.002***		*0.149*		*0.769*		*0.580*		*0.080*	
Hispanic	21	51.2	7	58.3	16	39.0	6	14.6	27	90.0	25	75.8	33	80.5	35	85.4
Non-Hispanic	83	59.3	19	39.6	53	36.6	57	40.1	108	96.4	93	73.2	122	84.1	136	93.8
**Immigration status**	*0.100*		*0.171*		*0.078*		***0.004***		*0.122*		*0.683*		*0.107*		*0.549*	
Citizen	61	54.0	13	36.1	38	32.2	35	30.2	82	96.5	78	73.6	96	81.4	111	94.1
All other	32	68.1	11	55.0	22	46.8	25	54.3	34	89.5	30	76.9	43	91.5	43	91.5
**Housing status**	*0.649*		*0.200*		*0.675*		*0.121*		*0.153*		*0.575*		***0.020***		***0.026***	
Stable	81	60.9	20	47.6	15	39.5	53	39.0	104	92.9	87	73.7	119	86.9	130	94.9
All other	21	56.7	5	29.4	49	35.8	9	25.0	21	100.0	22	68.7	27	71.0	32	84.2
**Insurance**	***0.025***		*0.437*		***<0.001***		*0.200*		*0.741*		*0.280*		*0.612*		*0.648*	
Private	27	46.5	13	48.1	10	16.4	25	41.0	44	93.6	36	67.9	52	85.2	57	93.4
All other	82	64.1	13	38.2	61	46.9	40	31.5	94	95.0	85	75.9	107	82.3	119	91.5
**Language**	*0.565*		*0.773*		***0.047***		***0.017***		*0.452*		*0.667*		*0.081*		*0.127*	
English	67	57.8	17	44.7	39	32.2	34	28.6	86	95.6	80	74.1	96	79.3	114	94.2
All other	41	62.1	9	40.9	31	47.0	30	46.1	50	92.6	39	70.9	59	89.4	58	87.9

^1^Bold indicates significant at alpha level of 0.05.

^2^STI testing included receipt of syphilis, chlamydia, and gonorrhea testing vs. did not receive ≥1 of these tests.

In order to further understand the relationship between Hispanic ethnicity, primary language, U.S. citizenship, and receipt of safe sex counseling, additional analyses were done (data not shown). Among English speakers, there were no differences in the proportion of women who had documented safe sex counseling between Hispanics and non-Hispanics. Among non-English speakers, Hispanics were much less likely than non-Hispanics to have safe sex counseling documented (12% vs. 65.9%, p < 0.001). A similar pattern was observed when stratified by U.S. citizenship. Among U.S. citizens, there were no significant differences between the proportion of Hispanics and non-Hispanics with documented safe sex counseling. Among non-citizens, Hispanics were much less likely than non-Hispanics to have safe sex counseling documented (8.3% vs. 68.6%, p < 0.001).

### Multivariable logistic regression models for receipt of STI testing and safe sex counseling

Documented receipt of all three recommended STI tests (syphilis, chlamydia, and gonorrhea) and documented receipt of safe sex counseling was related to a number of demographic factors among this patient population. Results of multivariable logistic regression models identifying factors that may be independently related to receipt of these services are shown in Table 4. We found that having private insurance significantly *decreased* a patient’s likelihood of documented receipt of all three STI tests in a multivariable logistic regression model that included race, Hispanic ethnicity, immigration status, housing status, health insurance status and language (OR: 0.20; 95% CI: 0.08 - 0.52). Women of Hispanic ethnicity were less likely to have documented receipt of safe sex counseling in a multi-variable logistic regression model that include race, immigration status, housing status, insurance status and language (OR: 0.26; 95% CI: 0.07 - 0.94).

**Table 4:** **Adjusted multivariable logistic regression models for receipt of STI testing and safe sex counseling**^**1**^

	**STI testing**^**2,3**^		**Safe sex counseling**^**3**^	
	**OR**	**95% CI**	**OR**	**95% CI**
**Race**	*p = 0.256*		*p = 0.177*	
White	.55	(.20-1.54)	.48	(.17-1.39)
**Ethnicity**	*p = 0.428*		***p = 0.040***	
Hispanic	.65	(.23-1.88)	.26	(.07-.94)
**Immigration status**	*p = 0.854*		*p = 0.548*	
Citizen	.89	(.24-3.22)	0.67	(.18-2.51)
**Housing status**	*p = 0.420*		*p = 0.134*	
Not stable	1.43	(.60-3.42)	2.12	(.79-5.68)
**Insurance**	***p = 0.001***		*p = 0.577*	
Private	.20	(.08-.52)	1.26	(.56-2.83)
**Language**	*p = 0.724*		*p = 0.450*	
English	.80	(.22-2.82)	.59	(.15-2.28)

^1^Bold indicates significant at alpha level of 0.05.

^2^STI testing included received syphilis, chlamydia, and gonorrhea testing vs. did not receive ≥1 of these tests.

^3^Adjusted for race, Hispanic, immigration status, housing status, insurance, and language.

## Discussion

Little is known about the provision of preventive health services to HIV positive women receiving care in an HIV/AIDS specialty clinic, despite evidence that such women may suffer disparities in conditions such as heart disease, diabetes, HPV infection, and some types of cancer [11,12,14-17]. This study is among the first to evaluate factors associated with receipt of preventive care among such women. We found that among all HIV positive women seen for HIV/AIDS care in 2009 in an academic specialty clinic, there were wide variations in the types of services they received. Notably, STI testing and safe sex counseling were documented in the medical records of only 37% and 33.9% of women, respectively. Documentation of cancer screening was also low, with 56.8% of women having documentation of cervical cancer screening, 65% having documentation of mammography where indicated, and only 10% having documentation of colorectal cancer screening where indicated. In contrast, in Utah in 2010, 71.8% of women age 50 and older received a mammogram, [34] 74% received cervical cancer screening, [35] and 66.3% received colorectal cancer screening [36]. Of note, nearly a third of patients in this study (31.8%) were covered solely through the Prevention Care Alliance (Ryan White Part C Program). Due to limited resources, such patients are not routinely referred for services such as dental or eye exams or colonoscopy. The majority of these patients do not have funds to cover the out of pocket expenses associated with these services.

In the context of the preventive health services received by this patient population, it is important to point out that these patients are clinically challenging for several reasons. More than one-third of the HIV positive women in this study spoke a language other than English, nearly 1 in 5 did not have stable housing arrangements, and there were high rates of mental health issues and substance abuse among them. These issues make it challenging for clinicians to provide a full range of preventive services during busy HIV/AIDS-related clinic visits, as many these women have multiple issues that need to be addressed in addition to their HIV/AIDS care. However, these challenges are likely true for any healthcare provider caring for HIV positive women and are not unique to HIV positive women in Utah.

Interestingly, we found that racial minorities and non-English speakers were *more* likely to receive some types of preventive services than Caucasian or English-speaking women. For example, we found that White women, U.S. citizens, and English-speakers were less likely to have safe sex counseling documented. White women and those with private health insurance were less likely to have STI testing documented. Women with private insurance were also less likely to have cervical cancer screening documented. In multivariable models, women with private health insurance were less likely to have documented STI testing. These findings may be due to several issues including the practices of specific providers who tend to care for different types of patients (English vs. Spanish-speaking patients, for example). In addition, the out-of-pocket expenses associated with some of these services may require co-payments for privately insured patients, but may be available at no cost to publically insured patients. Finally, privately insured patients may have primary health care providers outside of the University of Utah system and may not have preventive services received outside of the system documented in their University medical records.

This study advances prior research by incorporating data from a clinic which serves a large proportion of HIV positive women in the state of Utah. Clinic 1-A is the premier HIV-AIDS clinic in Utah, and captures the majority of HIV positive women in the state. A thorough chart audit process was used, including searching for lab values (i.e. cervical cancer screening results) if they were not included in the patient’s medical record. This process provided accurate data about the provision of preventive health services to all patients seen in the clinic in 2009. As the HIVMA introduced new guidelines for preventive care of HIV positive individuals in 2009, these data can be used in future studies to explore changes in providers’ recommendation and provision of preventive services for this population.

There are several important limitations to the study. The study clinic is not part of a closed health care system, and it is possible that some patients may have had primary care providers outside of the University of Utah system. We were unable to query those records. In many cases, the provision of clinical services was not documented at all. In these instances, we assumed that the services were not provided. This may be an inaccurate assumption, for example, in the case of safe-sex counseling, which may be done conversationally but not recorded in the medical record. We did not collect detailed information on contraceptive methods (e.g., tubal, hormonal etc.) as many women received these from other sources (OB/GYNs, family planning clinics). Investigating the provision of contraceptives to HIV positive women is an important future research opportunity.

Lastly, at the time of this study, the clinic was using primarily paper-based medical records, with some labs and other results available electronically. Since that time, the clinic has switched to exclusively electronic charting. In the future, we hope to compare data on the provision of preventive health services under the new electronic medical record system to see if utilization of such a system has helped improve reporting of the proportion of women receiving recommended services.

When reviewed in the context of the HIVMA/IDSA guidelines for the preventive care of HIV positive women, patients in this study were less likely to have received mammography, cervical cancer screening, or colonoscopy then women in the general Utah population. Receipt of colorectal cancer screening was notable low, with only 1 in 10 patients receiving such screening. Receipt of STI testing and safe sex counseling were also very low, with documentation in the medical records of 37% and 33.9% of patients, respectively. However, on a positive note, rates of substance abuse screening were high (91.7%) and nearly three quarters of women (71.9%) received an annual influenza vaccine.

## Conclusions

In 2009, the HIV Medicine Association (HIVMA) of the Infectious Diseases Society of America (IDSA) recommended HIV positive women receive routine, preventive health care as a part of their regular HIV/AIDS care. However, in an academic specialty HIV/AIDS clinic serving a large proportion of the HIV positive women in Utah, we found that the provision of many recommended preventive services was lacking, particularly among women with fewer socio-demographic risk factors (i.e., insured, White race, stable housing etc.). Our findings serve as a reminder that HIV/AIDS providers must focus on the needs of all women for preventive care services, not only those who may be perceived to be part of a vulnerable population. In addition, failure to provide preventive services such as STI testing, cancer screening, or safe sex counseling to all patients represents a missed opportunity for provision of services that are important from both a clinical and public health perspective. Finally, given the longevity of HIV positive patients in the era of HAART and the extensive list of recommended preventive health care services, the optimal setting for the care of such patients may need to shift away from infectious diseases specialty clinics towards general medicine clinics with HIV/AIDS consultants. As HIV/AIDS continues to evolve as a disease, new health care models may be needed to address the complex needs of patients including preventive health care, mental health and substance abuse services, and social services in addition to HIV/AIDS care.

## Consent

As this was a medical record review and no patients were contacted, informed consent was waived by the University of Utah Institutional Review Board.

## Abbreviations

HAART: Highly Active Anti-Retroviral Therapy; HIVMA: HIV Medicine Association; IDSA: Infectious Diseases Society of America; USPSTF: United States Preventive Services Task Force; HIV: Human Immunodeficiency Virus; AIDS: Acquired Immune Deficiency Syndrome; STI: Sexually Transmitted Infection; Pap: Papanicolaou test; HPV: Human Papillomavirus; TB: Tuberculosis; Clinic 1A: University of Utah Infectious Diseases Clinic.

## Competing interests

The authors declare that they have no competing interests.

## Authors’ contributions

SES designed the study, assisted with the chart reviews, assisted with the data analysis, and drafted the manuscript. DK and EW performed data analysis and drafted the manuscript. JT, MS, and KR provided input into the study design, assisted with the chart reviews, and provided critical feedback on manuscript drafts. All authors read and approved the final manuscript.

## Pre-publication history

The pre-publication history for this paper can be accessed here:

http://www.biomedcentral.com/1472-6874/14/37/prepub
